# Donor-derived del[20q] following allogeneic-hematopoietic cell transplantation: a case with 26-year follow-up and literature review

**DOI:** 10.1038/s41409-026-02801-8

**Published:** 2026-02-14

**Authors:** Clara Bouley, Min Fang, Jerald Radich, Cecilia CS Yeung, Judy Campbell, Kate Kroeger, Lan Beppu, Marco Mielcarek, Rainer Storb, Masumi Ueda Oshima

**Affiliations:** 1 University of Washington School of Medicine, Seattle, WA USA; 2 Fred Hutchinson Cancer Center, Seattle, WA USA

**Keywords:** Oncogenesis, Stem-cell research, Myelodysplastic syndrome

## Abstract

Donor-derived cytogenetic abnormalities are a rare finding following allogeneic hematopoietic cell transplantation. Deletion of the long arm of chromosome 20 [del(20q)] is one of the more frequently observed structural abnormalities, but its significance in the post-transplant setting remains unclear. We describe a unique case of donor-derived del(20q) with 26 years of post-transplant follow-up, the longest reported to date. The recipient remains well with normal blood counts despite persistent del(20q) in both myeloid and lymphoid lineages and the presence of coexisting somatic mutations in *DNMT3A* and *TP53*. Retrospective analysis of the donor’s marrow confirmed del(20q) and low-level *DNMT3A* and *TP53* mutations at the time of transplant; the donor later developed therapy-related MDS after radiation therapy for thyroid cancer. To contextualize this case, we reviewed 20 published reports of donor-derived del(20q) post-transplant. The median time to detection was 16 months post-transplant, and 35% of cases progressed to donor-derived malignancy. Among those who progressed, the median time to malignancy diagnosis was 22 months post-transplant. Clinical outcomes across cases ranged from asymptomatic persistence and cytopenias to donor-derived myeloid malignancies, highlighting the need for long-term follow-up and potential use of molecular profiling to better define the neoplastic potential of donor-derived del(20q) after transplantation.

## Introduction

Clonal cytogenetic abnormalities can be transferred from donor to recipient following allogenic hematopoietic cell transplantation (allo-HCT), in which abnormal clones repopulate the marrow. Among donor-derived clonal cytogenetic abnormalities observed post-transplant, monosomy 7, gain of 1q, and deletion of 20q (del[20q]) are among the most frequently detected and clinically described abnormalities [1, 2].

Deletion of the long arm of chromosome 20 [del[20q]] is one of the most common large structural genetic mosaicism abnormalities detected in human leukocytes [3, 4]. While frequently associated with hematologic cancer, del[20q] can exist in healthy individuals and is frequently observed in older adults with a prevalence exceeding that of myeloid malignancies [4]. It is typically classified as a clonal cytogenetic abnormality of undetermined significance and is not a myelodysplastic syndrome (MDS)–defining finding in the absence of morphologic dysplasia [2, 5]. While often benign, it is proposed that del[20q] may serve as a “first hit” in leukemogenesis, especially in the presence of cooperating mutations [6, 7].

Given the association between aging and clonal hematopoiesis, structural mosaic events like del[20q] may be inadvertently transferred from older donors during allo-HCT [1, 2, 4]. While donor origin is typically inferred from chimerism studies, few reports have confirmed del[20q] in donor material—either through retrospective analysis or subsequent donor diagnosis following detection in recipient [8–10]. Documented outcomes of donor-derived del[20q] range from stable persistence to progression to MDS or acute myeloid leukemia (AML) [1, 2, 10, 11]. Interpretation remains complicated by limited longitudinal follow-up and incomplete donor evaluation in most published cases.

Here, we present the longest documented follow-up of a recipient of donor-derived del[20q]. We also review all reported cases of donor-derived del[20q] following allo-HCT, representing the largest collection to date. Together, this case and literature review refine the clinical understanding of donor-derived del[20q], highlighting its variable course, challenges in early classification, and the importance of long-term recipient surveillance.

## Materials and methods

### Ethics approval and consent to participate

Subject enrollment and testing of specimens were approved through Fred Hutchinson Cancer Center Institutional Review Board (Protocol #9787). The subject signed informed consent. All methods were performed in accordance with the relevant guidelines and regulations.

### Case report methods

#### Sample collection and processing

Twenty milliliters (mL) of recipient peripheral blood were collected at each of the two timepoints (20 and 26 years post-transplant). Approximately 10 mL of fresh sample was sent for fluorescent in situ hybridization (FISH) studies. From the remainder of the sample, peripheral blood mononuclear cells (PBMC) were isolated and were frozen in fetal bovine serum (FBS) with 10% dimethyl sulfoxide and stored in liquid nitrogen. All laboratory testing was performed once per time point given limited sample quantity.

#### Fluorescent in situ hybridization (FISH)

FISH was performed using a locus-specific DNA-probe for 20q12 on flow-sorted myeloid and lymphoid cell populations labeled with fluorochrome-conjugated antibodies. Through the initial seven-years post-transplant, a single-color probe construct was used (Vysis, Downers Grove, IL). Beyond this time period, a dual-color probe targeting 20q12 and 20q13.1 (control) was used (LPH020 from Cytocell, Cambridge, UK). At least 100 interphase nuclei per sorted population were analyzed. Cells with two 20q12 signals were counted as “normal” and cells with one 20q12 signal, as “del[20q].”

#### Next-generation sequencing

After slow thawing of cryopreserved PBMC in Roswell Park Memorial Institute medium with 10% FBS, DNA was extracted using the QIAamp DNA Blood Mini Kit (Qiagen) and quantified with Qubit. Next generation sequencing was performed with the Oncomine Myeloid MRD Assay (Thermo Fisher Scientific).

### Literature review methods

We conducted a targeted literature review to identify published cases of donor-derived del[20q] after allo-HCT. PubMed was searched as the primary database, followed by Google Scholar to capture additional reports not indexed in PubMed. Duplicate records were excluded. Inclusion criteria were: (1) complete and confirmed donor chimerism, and (2) del[20q] detection above standard thresholds by either conventional cytogenetics ( ≥ 2/20 metaphases) or fluorescence in situ hybridization (FISH; ≥4%). Sequential reports describing earlier follow-up of the present case were counted once to avoid duplication [9, 10]. A PRISMA flow diagram summarizing the search strategy and study selection process is presented in the Results section, and complete search queries by database are provided in Supplemental Table 1.

### Case report

**Fig. 1:**
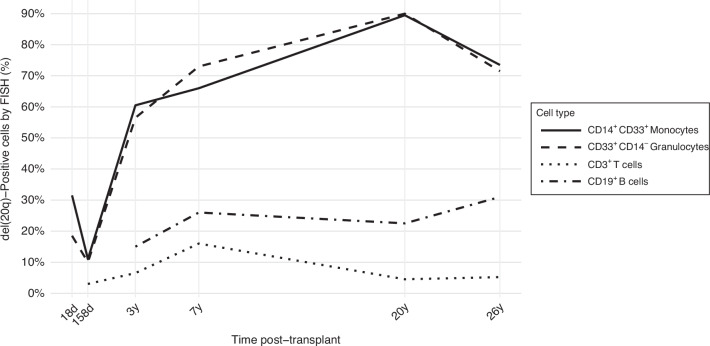
Percentage of del20q)-positive cells by FISH in sorted peripheral blood cells spanning 26 years post-transplant by cell type. FISH Fluorescent in situ hybridization, d days, y years.

A 41-year-old woman with refractory angioimmunoblastic T-cell lymphoma underwent allo-HCT from her 50-year-old HLA-identical sister [9]. The donor had mild anemia (hematocrit 32–36%) and borderline thrombocytopenia (platelet count 140-200 × 109/L) but additional workup was initially deferred. The peripheral blood CD34 cell yield from the donor after 5 days of growth factor mobilization was insufficient; thus, donor marrow harvest was pursued. Donor marrow evaluation showed left-shifted myelopoiesis with hypergranulated myeloid cells but no increase in blasts or overt dysplasia; cytogenetic testing was therefore performed and revealed del[20q] in 3 of 20 metaphases, concerning for an early clonal process. The recipient achieved rapid neutrophil and platelet engraftment following myeloablative conditioning and transplant, with full donor chimerism in lymphoid and myeloid lineages by day 34 post-transplant [10]. Del[20q] was detectable at low levels in the recipient blood by FISH on days 18 and 158 post-transplant. Follow-up at 3- and 7-years post-transplant showed increase in the del[20q] population in all cell types (Fig. 1). While not detected at engraftment, del[20q] appeared in T cells at day 158, and in B cells beginning at 3 years. The recipient was treated with cyclosporine, beclomethasone and prednisone for chronic graft-versus-host disease (GVHD) of the mouth, skin and upper GI tract at around three months post-transplant but had no further complications. The recipient also had intermittent thrombocytopenia and mild normocytic anemia persisting at 8 years post-transplant [10] with no apparent clinical sequalae and had a normal complete blood count at the time of last contact at 26 years post-transplant.

Subsequent sampling and FISH analysis of the recipient’s blood has now been performed 20- and 26-years post-transplant. The fraction of del[20q]-positive cells remained dominant ( > 70%) in myeloid fractions and persisted at lower levels in lymphoid cells (Fig. 1). Next-generation sequencing of donor’s archived marrow and recipient blood at 20- and 26-years post-transplant revealed low-level variants ( < 2% variant allele frequency) in DNA methyltransferase 3 alpha (*DNMT3A*) and tumor protein p53 (*TP53*) (Table 1). Remarkably, the patient has remained in remission with a normal complete blood count. However, in noteworthy contrast, the donor developed progressive MDS after radiation treatment for thyroid cancer and died from complications of MDS treatment, 20 years after serving as the donor.

**Table 1:** Variants detected in the donor marrow at HCT and in the recipient blood cells at 20 and 26 years post-HCT.

Gene	Transcript effect	Variant consequence	ClinVar database significance	Donor baseline DNA at HCT, AF (%)	Recipient 20 years post-HCT, AF (%)	Recipient 26 years post-HCT, AF (%)
*DNMT3A*	p.Pro904Leu	missense	pathogenic/likely pathogenic	0.65	0.56	0.09
*TP53*	p.Arg273Cys	missense	pathogenic/likely pathogenic	1.36	1.5	0.05
*TP53*	p.Met237Ile	missense	pathogenic/likely pathogenic	0.11	0.15	not called
*TP53*	p.Tyr234His	missense	pathogenic/likely pathogenic	0.18	0.16	not called
*TP53*	p.Tyr220Cys	missense	pathogenic	0.11	0.09	0.23

*DNMT3A* DNA methyltransferase 3 alpha, *TP53* tumor protein p53, *AF* allelic frequency.

### Literature review

**Fig. 2:**
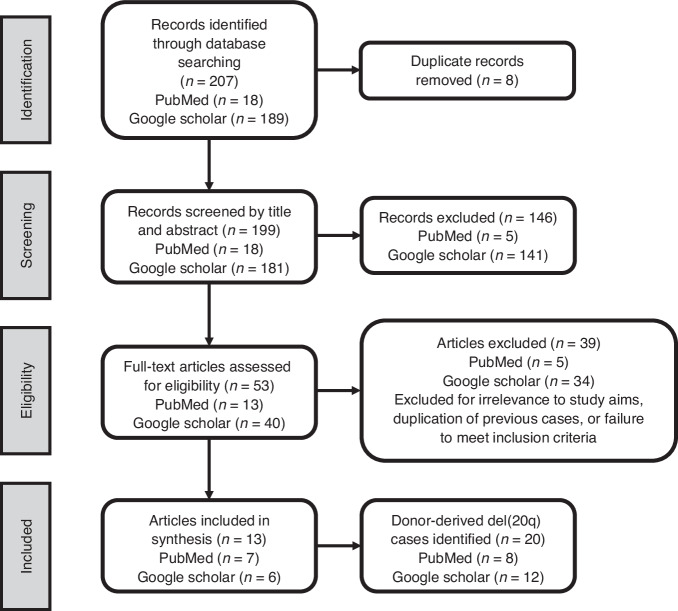
PRISMA (Preferred Reporting Items for Systematic reviews and Meta-Analyses) flow diagram for identifying articles reporting donor-derived del(20q) following allo-HCT.

We identified 20 unique cases of donor-derived del[20q] following allo-HCT across 13 publications (Fig. 2; Table 2) [1, 2, 6, 8–18]. All cases met inclusion criteria requiring confirmed complete donor chimerism and detection of del[20q] above accepted cytogenetic or FISH thresholds. As described in the Methods, prior reports of our present case were counted once to avoid duplication [9, 10]. Figure 2 outlines the search and selection process, including database source and exclusion rationale.

**Table 2:** Clinical and cytogenetic features of reported cases of donor-derived del(20q) following allogeneic hematopoietic cell transplantation.

Case Number	Reference	Year	Recipient age/sex	Time to detection of del(20q) (months), recipient, post-HCT	Follow-up duration (months)/ alive at last follow-up (Y/N)	Recipient karyotype, pre-HCT	Recipient karyotype, post-HCT	Donor-derived myeloid malignancy, time post-HCT (months)	Mutations detected post-HCT
1 (present case)	(10,11)	1999, 2006	41/F	0.6	319/Y	ND	del(20q) / 18.5- 31.5% FISH	n/a	*DNMT3A, TP53*
2	(13)	2005	36/M	7	9/N	46, XY, der(2) t(2;8) (p16; q21), del(6)(q21q23), del(8)(p12), −13, +21, +mar[cp28]	46 XY, del (20)(q12∼13) [11]/46 XY [12]	MDS, 7	ND
3	(12)	2006	45/M	36	48/Y	ND	46, XX, del(20q) / ND	n/a	ND
4	(14)	2011	58/M	22	24/Y	46,XY,t(11;14)(q13;q32)	(46, XX, del(20), (q11.2q13.1)[8]//46, XX[12])	MDS, 22	ND
5	(15)	2013	63/M	16	26/Y	46,XY[20]	46,XX,del(20)(q11.2q13.3)[2]/ 46,XX[18]	MDS, 26	ND
6	(15)	2013	62/M	48	78/Y	46,XY,del(6)(p23),der(9)t(9;22;19) (q34;q11.2;p13.1),der(22)t(9;22) (q34;q11.2)[20]	46,XX,del(20)(q11q13)[2]/ 46,XX[18]	n/a	ND
7	(9)	2015	57/M	5	12/N	46,XY[23]	46,XX,del(20)(q11.2)[13]/46,XX[17])	n/a	ND
8	(16)	2019	57/M	1	30/Y	46,XY,der(5)t(5;12) (q31;q13),-12,del(20)(q11.2q13.3),+mar[3]/46,XY[1]	del(20q) / 2/20 metaphases	n/a	ND
9	(17)	2020	65/F	23	29/N	Female complex karyotype (no detailed info)	46,XY,der(2)t(2;3)(p21;q26.2),der(3)t(3;3)(q10;q10)add(3)(q26.2)t(2;3), del(20)(q11.2q13.3)[13]	AML, 16	*BCOR, FLT3, PIGA*
10	(18)	2020	64/M	3.3	24/Y	46,XY [20]	46, XX [20] / 11.6% FISH (day 100), 46,XX, del (20)(q11.2q13.3) [10]/46XX[10] / 41.4% FISH (2 years)	n/a	ND
11	(18)	2020	64/M	3.3	30/Y	46,XY [20]	46,XY [19]/46,XY del (20q)[1] / 6.2% FISH	n/a	ND
12	(18)	2020	60/M	42	42/Y	46,XY [20]	46,XY, del (20q)[3]/46,XY [27]	n/a	ND
13	(2)	2021	30/M	47	132/Y	47,XY, 8[3]/ + 47,XY,idem,del(21)(q22)[15]	46,XX,del(20)(q11.2q13.3)[3]/46,XX[17]	n/a	No mutations detected
14	(2)	2021	19/F	24	72/Y	46,XX,t(8;16)(p11.2;p13.3), inv(9)(p12q13)[20]	46,XY,del(20)(q11.2q13.1)[7]/46,XY[13]	n/a	No mutations detected
15	(7)	2021	49/M	72	72/N	46,XY,t(6;11)(q27;q23)[18]/ 46,XY[2]	46,XX,del(20)(q11.2q13.3) [4]/46,XX[16]	AML/myeloid sarcoma, 72	No mutations detected
16	(19)	2023	55/M	1	24/Y	46, XY [20]	45,XY,dic(18;20) (p11.2;q11.2)[5]/46,XY[15] / del(20q) quantification via FISH	n/a	No mutations detected
17	(1)	2023	ND	89	91/Y	ND	del(20q), +21, + 22/ND	AML, 89	*MPL, NF1, RUNX1*
18	(1)	2023	ND	12	18/N	ND	del(20q)/ND	n/a	ND
19	(1)	2023	ND	16	170/Y	ND	del(20q)/ND	n/a	ND
20	(1)	2023	ND	1.8	142/Y	ND	del(20q)	MDS, 18	ND
**All cases (n** = **20)**	(1,2,7,9–19)		**Median** = **57, Range** = **19-65**	**Median** = **16, Range** = **0.6–89**	**Median** = **36, Range** = **9–319**				
***Malignant cases (n*** = **7), (7/20** = **35%)**	(1,7,13–15,17)		**Median** = **58, Range** = **36-65**	**Median** = **22, Range** = **1.8-89**	**Median** = **29, Range** = **9–142**			**AML (n** = **3), MDS (n** = **4) Median** = **22, Range** = **7-89**	

“No mutations detected” indicates sequencing performed but no pathogenic variants identified. *HCT* hematopoietic cell transplantation; *ND* no data available, *n/a* not applicable.

Recipient characteristics are summarized in Table 2. The median recipient age was 57 years (range, 19–65), with 81% being male. Most patients had HLA-identical sibling donors, and AML was the most common primary disease (*n* = 6), followed by CML and CLL (*n *= 2 each). The median time to detection of del[20q] was 16 months post-transplant (range, 18 days–89 months), and the median follow-up was 36 months (range, 9–319 months). Sequencing results were reported in 7 (35%) cases (including present case). Among these, mutations in myeloid malignancy-associated genes were identified in three recipients post-transplant, while four recipients had no mutations detected (Table 2).

A total of 7 cases (35%) documented progression to a donor-derived myeloid malignancy, most commonly MDS (*n* = 4) and AML (*n* = 3) (Table 2; Supplemental Table 2) [1, 6, 12–14, 16]. The median age of these recipients was 58 years (range, 36–65), time to detection of del[20q] was 22 months post-transplant (range, 1.8–89), and follow-up duration was 29 months (range, 9–142). The median time to diagnosis of donor-derived malignancy post-transplant was 22 months (range, 7–89). Del[20q] was identified prior to malignancy diagnosis in two cases, concurrently in four, and after diagnosis in one. Among the three cases of donor-derived malignancy with available sequencing data, one had no detectable mutations, one harbored *BCOR*, *FLT3*, and *PIGA* mutations, and one showed *MPL, NF1*, and *RUNX1* mutations (Table 2).

Five deaths occurred overall, three among recipients with donor-derived malignancy [1, 6, 8, 12, 16]. Among the 7 cases with donor follow-up, 4 had confirmed del[20q] in donor cells—either retrospectively or through subsequent donor testing—while 2 had normal donor karyotypes and 1 lacked cytogenetic data (Supplemental Table 2) [1, 8–11, 14, 18]. Clinical manifestations in recipients varied across cases but most commonly included thrombocytopenia, anemia, GVHD, and other cytopenia or dysplastic features on bone marrow morphology. Detailed post-transplant clinical courses and donor characteristics are provided in Supplemental Table 2.

## Discussion

We describe the longest-known case of persistent donor-derived del[20q], with 26 years of clinical follow-up and retrospective donor sample analysis from the time of transplant. This case is presented alongside a review of all other reported instances of donor-derived del[20q] after allo-HCT, representing the most comprehensive synthesis to date.

This case is unique in its longitudinal scope and molecular description, as well as the starkly divergent outcomes between donor and recipient. From shared hematopoietic origins, the donor and recipient experienced very different clinical trajectories: the recipient has remained in remission with normal blood counts despite a dominating del[20q] clone in myeloid cells and multiple pathogenic/potentially pathogenic mutations in *TP53*, while the donor experienced progressive MDS following radiation exposure. The contrasting outcome in the donor highlights how similar clonal architectures can evolve differently depending on subsequent environmental exposures, immune context, marrow microenvironment and time. The benign course of the recipient is consistent with prior observations comparing somatic mutation frequency in the blood cells of donor-recipient pairs surviving decades after allo-HCT [19]. This prior study did not show substantial expansion of donor-derived somatic mutations in recipients, even after several decades [19].

Across the 20 cases identified in the literature, donor-derived del[20q] displayed highly variable clinical outcomes, ranging from asymptomatic persistence to donor-derived myeloid malignancies [1, 2, 6, 8–18]. Notably, four of the seven donor-derived malignancy cases had del[20q] detected at the time of malignancy diagnosis, two were identified prior, and one was detected afterward. The median time to both del[20q] detection and malignancy onset was 22 months post-transplant, suggesting that del[20q] may remain subclinical until malignant transformation occurs. These patterns suggest that earlier and more consistent surveillance may be warranted.

Although sequencing data were available in a subset of cases reviewed, the number of cases remains too limited to draw conclusions about how co-occurring oncogenic mutations may influence the malignant evolution of del[20q] clones in the post-transplant setting. Interpretation is further complicated by incomplete reporting and frequent reliance on sex-mismatched transplants to confirm donor origin, which may introduce reporting bias. The rarity of del[20q] in post-allo-HCT recipients is reflected in its reported prevalence of just 0.15–0.45% across two institutional reviews [1, 2]. Our case demonstrates that del[20q], though classically considered a “first hit” in leukemogenesis, can persist for decades without clinical consequence, even in the presence of known pathogenic mutations in *TP53* and *DNMT3A* [6, 7].

Our case also raises the question of whether cytogenetic testing should be incorporated into routine donor evaluation prior to allo-HCT. At our center, screening for donor cytogenetic abnormality or clonal hematopoiesis is not currently routinely performed. Donors are assessed based on standard clinical and laboratory criteria. Large-cohort studies of donor clonal hematopoiesis suggest that mutations associated with clonal hematopoiesis of aging (e.g., *DNMT3A*, *TET2*) generally do not worsen recipient outcomes and may, in some cases, enhance graft-versus-leukemia effects through altered immune signaling, whereas other mutations in high-risk genes (e.g., *TP53*, splicing factors) are linked to donor cell leukemia [20]. These observations as well as our case highlight the genetic and clinical heterogeneity of donor-derived clonal processes post-transplant. Donors harboring known clonal or cytogenetic abnormalities may still be suitable after individualized risk-benefit assessment that considers recipient disease urgency for allo-HCT and availability of alternative donor options. Further study is needed to develop evidence-based guidance on appropriate donor evaluation for clonal hematopoiesis and cytogenetic abnormalities. If donors harboring such abnormalities are to be used, evidence to guide the approach to post-transplant monitoring in the recipient is needed.

In conclusion, donor-derived del[20q] displays a wide spectrum of outcomes, from stable, benign persistence to progression into donor-derived myeloid malignancies. The current case is unique in its long-term follow-up of 26 years of clinically benign but sustained del[20q]-predominant myeloid hematopoiesis despite coexisting mutations in genes implicated in hematologic malignancy. Future studies with extended follow-up, comprehensive donor-recipient data, and molecular characterization are needed to better define the behavior of donor-derived del[20q] after allo-HCT. Identifying the mutational burden and specific co-mutations that may elevate the risk of malignant transformation in the post-transplant setting remains an important area of further investigation.

## Supplementary information

Supplemental Table Legends

Supplemental Table 1

Supplemental Table 2

## Data Availability

All data generated or analyzed during this study are included in this published article and its supplementary information files.
