# Supplementary material for: Donor-derived del[20q] following allogeneic-hematopoietic cell transplantation: a case with 26-year follow-up and literature review
Source: Bone Marrow Transplant. 2026 Feb 14;61(4):445–51. doi: 10.1038/s41409-026-02801-8 (PMC13056576; doi:10.1038/s41409-026-02801-8)
Supplement: Supplementary file 1 — Supplemental Table Legends [file 41409_2026_2801_MOESM1_ESM.docx]

Supplemental Table 1. Search queries used to identify cases of donor-derived del(20q) following allo-HCT. Final search date: June 17, 2025.

Supplemental Table 2: Detailed post-transplant clinical courses and donor characteristics. *related to second transplant from donor one, **related to follow-up transplant from donor two; HCT, hematopoietic cell transplantation; AITL, angioimmunoblastic T-cell lymphoma; CML, chronic myelogenous leukemia; MCL, mantle cell lymphoma; MDS, myelodysplastic syndrome; U, unclassified; CLL, chronic lymphocytic leukemia; AML, acute myeloid leukemia; SLL, small lymphocytic leukemia; THRBCL, T-cell/histiocyte-rich large B-cell lymphoma; t-AML, therapy-related AML; GVHD, graft-vs-host disease; TBI, total body irradiation; ATG, anti-thymocyte globulin; VP-16, etoposide; CMV, cytomegalovirus; EBV, Epstein-Barr virus; GI, gastrointestinal; F, female; M, male; ND, no data; y, years; mo, months.
